# Expression of bluetongue virus core proteins VP2 and VP7 by bovine herpesvirus-4 confers protection against virulent challenge in a mouse model

**DOI:** 10.1186/s13567-026-01771-5

**Published:** 2026-05-28

**Authors:** Valentina Franceschi, Pablo Nogales-Altozano, José M. Rojas, Sergio Minesso, Noemí Sevilla, Gaetano Donofrio, Verónica Martín

**Affiliations:** 1 Centro de Investigación en Sanidad Animal (CISA)-Instituto Nacional de Investigación y Tecnología Agraria y Alimentaria (INIA)-Consejo Superior de Investigaciones Científicas (CSIC), Valdeolmos, 28130 Madrid, Spain; 2 Department of Veterinary Science, University of Parma, via del Taglio 10, 43126 Parma, Italy

**Keywords:** Vaccines, BTV, bovine-herpes-based vector, immune response, IFNAR^−/−^ mice

## Abstract

Bluetongue virus (BTV) remains a major threat to livestock health and agricultural economies worldwide due to its multiple serotypes, vector transmission, and wide geographic distribution. Vaccination is essential for controlling BTV outbreaks; however, developing vaccines that provide broad protection while complying with the requirements of differentiating infected from vaccinated animals (DIVA) remains challenging. In this study, we evaluated the antigenicity and protective efficacy of two BTV proteins, VP2 and VP7, delivered using a bovine herpesvirus-4 (BoHV-4)-based viral vector. Groups of mice lacking interferon-α/β receptor (IFNAR^−/−^) (C57BL/6 background) were immunized using a prime/boost regimen with BoHV-4 vectors expressing VP2, VP7, or both antigens. All vaccinated groups developed antigen-specific humoral and cellular immune responses. Following challenge with virulent BTV-8, complete protection was observed in the VP2 and VP7 single-antigen groups, while 80% protection was achieved with the combined VP2 + VP7 vaccination. Total anti-BTV IgG levels increased after booster immunization, with the highest pre-challenge titers detected in the combined vaccination group. After challenge, IgG levels rose further in VP2-immunized mice, whereas VP7-immunized animals showed comparatively lower titers. All vaccinated mice mounted BTV-specific cellular responses, as demonstrated by interferon (IFN)-γ enzyme-linked immunoadsorption assay (ELISPOT) and intracellular cytokine staining. T cells expressing IFN-γ, IL-2, TNF-α, or CD107a were detected, with evidence of polyfunctional responses across groups. Notably, VP7 immunization induced stronger cellular responses, including increased cytotoxic potential in CD4^+^ and CD8^+^ T cells. These findings demonstrate that BoHV-4 vectors expressing VP2 or VP7 elicit robust humoral and cellular immunity and confer protection against BTV, highlighting their potential as a versatile vaccine platform.

**Supplementary Information:**

The online version contains supplementary material available at 10.1186/s13567-026-01771-5.

## Introduction

Bluetongue virus (BTV) is a significant arthropod-borne pathogen that affects ruminants worldwide. It is transmitted through the bite of various species of midges belonging to the *Culicoides* genus. BTV is a member of the *Orbivirus* genus within the *Sedoreoviridae* family. The virus possesses a segmented double-stranded RNA (dsRNA) genome, and over 30 distinct serotypes are currently classified within the BTV serogroup. Of these, serotypes 1–24 are notifiable to the World Organization for Animal Health (WOAH). The viral genome encodes seven structural proteins and five nonstructural proteins. Among them, VP2 and VP7, used in this study, are particularly important [1]. VP2, the most variable protein, defines the serotype and mediates viral entry by facilitating host cell attachment alongside VP5. It is also the main inducer of neutralizing antibodies [2, 3]. VP7, a highly conserved core protein across serotypes, is considered a strong candidate for multiserotype vaccine development [4].

BTV is the causal agent of bluetongue disease (BT), an illness with major economic implications for the livestock industry. It primarily affects domestic and wild ruminants, such as sheep, goats, cattle, and deer [5], and occasionally could affect carnivores and camels [6]. Clinical signs include fever, inflammation, and hemorrhages, often resulting in high morbidity and mortality—particularly in sheep, where disease is more severe than in cattle, which typically show mild or no clinical signs [7]. Beyond its clinical effects, BTV poses a serious challenge to international trade due to livestock movement restrictions. BT is endemic to tropical and temperate regions [8], but due to global climate changes, both the virus and its vectors have expanded their range into central Europe. In September 2023, after more than a decade of being officially BT-free, the Netherlands reported the emergence of a highly virulent BTV serotype 3 (BTV-3) variant [9]. Despite vaccination efforts, the virus re-emerged in July 2024 and rapidly spread across much of Europe—including Germany, Belgium, France, Spain, and the UK [10]. In October 2024, BTV-12, previously unreported in Europe, was also detected in the Netherlands [11]. The consecutive emergence of novel serotypes highlights the urgent need for robust surveillance and innovative vaccine strategies.

In response to the challenges posed by BTV, extensive research efforts have been directed toward approaches that could improve current vaccine strategies. Vaccination remains a cornerstone in the control and prevention of BT, offering a proactive approach to mitigate the spread and impact of the virus. Various vaccine strategies have been explored, each with distinct advantages and limitations. Inactivated vaccines are the most widely used due to their safety profile compared to live-attenuated counterparts, which pose risks of reversion to virulence, reassortment, and reproductive issues [12–14]. However, inactivated vaccines generally show limited cross-protection across serotypes and lack capabilities for differentiating infected from vaccinated animals (DIVA).

Vectored recombinant vaccines are being touted as the safest DIVA new-generation vaccines [15–17]. Adenovirus-based vectors (Ad5) expressing BTV proteins such as VP2 or VP7 have shown the ability to induce both humoral and cellular immune protective responses in mice and sheep. Protection via VP7, in the absence of neutralizing antibodies, is associated with strong CD8^+^ T-cell responses [17]. Poxvirus-based vectors, such as modified vaccinia ankara (MVA), expressing VP2/VP5 and VP7 proteins, have also been effective in inducing protective immunity in murine and ovine models, eliciting humoral and cellular responses against homologous BTV challenge [18–21]. Other systems, such as capripoxvirus or canarypox expressing VP2 and VP7, have shown variable levels of success in sheep [20, 22]. Prime-boost strategies combining adenovirus (Ad) and MVA vectors expressing NS1 have demonstrated cross-serotype protection through conserved CD8^+^ T-cell epitopes [23]. In contrast, Bouet-Cararo et al. found that vectors expressing VP7 alone were insufficient to confer protection in sheep [24]. Reverse genetics has further advanced BTV vaccine development [25, 26] by generating live-attenuated viruses with deletions in NS3/NS3a or VP6, effectively blocking transmission or replication in vaccinated animals [27–29], in addition to advancing our understanding of virus biology [30]. Immunoinformatics tools have recently been applied to design broad-spectrum multi-epitope subunit vaccines, incorporating highly conserved BTV epitopes with strong predicted MHC binding and TLR activation potential [31, 32]. These approaches illustrate the ongoing efforts to improve current BTV vaccines.

One particularly promising avenue involves the use of Bovine herpesvirus-4 (BoHV-4) vectors as a platform for immunization against BTV or other relevant animal health pathogens. BoHV-4 vectors offer advantages such as high antigen capacity, strong immunogenicity, and genomic stability without integration. A nonpathogenic BoHV-4 strain [33, 34] has also shown safety on immunocompromised mice [lacking interferon-α/β receptor (IFNAR^−/−^) model] [35]. Similar to other herpesviruses, BoHV-4 establishes persistent infections in its natural host and in experimental hosts such as rabbits. Although viral DNA has been detected in numerous tissues during persistent infection by polymerase chain reaction (PCR), in situ hybridization, or virus recovery following explant culture, accumulated evidence indicates that cells of the monocyte/macrophage lineage represent a key site of persistence in both natural and experimental hosts. In contrast, when non-natural host species (swine, sheep, goat, rat, and mouse) are experimentally infected, BoHV-4 persistence has not been observed. Moreover BoHV-4 persistence could be abrogated by the deletion of open reading frame (ORF) 73 as previously described by Thirion et al. [36]. Thus, persistence should not be considered a safety issue. Moreover, BoHV-4 is a promising vaccine platform in veterinary medicine since it is capable of inducing, for instance, strong specific immune response when expressing different viral antigens against peste des petits ruminants virus (PPRV), in mice and sheep [37, 38], which conferred protection in sheep against a virulent PPRV challenge [38]. Previous work showed that recombinant BoHV-4 expressing BTV-8-VP2 induced neutralizing antibodies and reduced viremia in preclinical studies, but protection against BTV-8 was limited to prolonged survival [39].

In the present study, we decided to evaluate the antigenicity and protective efficacy of BTV VP2 and VP7 proteins expressed using a BoHV-4 viral vector as a means to improve vaccine efficacy. Our findings provide valuable insight into the potential of BoHV-4-based vaccines for combating bluetongue, contributing to the development of innovative and effective vaccine strategies for animal health.

## Materials and methods

### Cells and viruses

Human embryonic kidney cells(HEK) 293 T (ATCC: CRL-11268), bovine embryo kidney (BEK; Istituto Zooprofilattico Sperimentale, Brescia, Italy; BS CL-94), and BEK cells expressing *cre* (BEKcre) recombinase [40] were grown in complete Eagle’s minimal essential medium (cEMEM: 1 mM of sodium pyruvate, 2 mM of L-glutamine, 100 IU/mL of penicillin, 100 μg/mL of streptomycin, and 0.25 μg/mL of amphotericin B), supplemented with 10% fetal bovine serum (FBS). Vero cells (ATCC CCL-81) and baby hamster kidney (BHK-21, ATCC CCL-10) cells were grown in Dulbecco’ modified Eagle’s medium (DMEM), supplemented with 10% FBS. BTV serotype 8 (Belgium/06; MN837962 Gene Bank accession number) was used in all the experiments. BTV stocks were prepared as described previously [41]. Briefly, BHK cells were infected with BTV at a multiplicity of infection (MOI) of 0.1, and supernatants were collected at 48 hours post-infection (hpi). After three freeze/thaw cycles and 6 min sonication steps, the supernatants were clarified and stored at −80 °C until use. Virus titrations were performed in the semisolid agar medium using Vero cells as previously described [42]. BTV-8 stocks were purified through a 40% saccharose–Tris–NaCl–ethylenediaminetetraacetic acid (TNE; 10 mM Tris, 100 mM NaCl, 1.5 mM EDTA; pH 7.4) cushion by centrifugation in a SW28 rotor at 120 000 × *g* for 1 h at 4 °C. Recombinant BoHV-4A-ΔTK expressing VP2 BTV-8 protein (BoHV-4-A-CMV-IgK-VP2gDtm) and BoHV-4A-ΔTK were previously described [39].

### Plasmid generation

The nucleotide sequence coding for the BTV-8 VP7 ORF (Belgium/06) was chemically synthesized (Genscript) and subcloned in a BoHV-4 shuttle vector.

After the chemical synthesis VP7 ORF was labelled with the hemagglutinin (HA) tag via a PCR reaction, using a couple of primers, providing an XbaI site at the 5′ of the ORF and the HA tag, followed by a BamHI site at the carboxyterminal (XbaI-VP7-sense: 5′-CCCCTCTAGACCACCATGGACACTATCGCTGCAAGAGCAC-3′; BamHI-VP7-HA antisense: 5′-CCCCGGATCCTTAGGCGTAGTCGGGCACGTCGTAGGGGTACACATAGGCGGCGCGTGCAATAGCACG-3′).

The PCR amplification reaction was carried out in a final volume of 50 μL, containing 20 mM Tris–hydrochloride pH 8.8, 2 mM MgSO_4_, 10 mM KCl, 10 mM (NH_4_)_2_SO_4_, 0.1 mg/mL bovine serum albumin (BSA), 0.1% (volume/volume [v/v]) Triton X-100, 5% dimethyl sulfoxide (DMSO), 0.2 mM deoxynucleotide triphosphate, and 0.25 μM of each primer. Pfu recombinant DNA polymerase 1U (Thermo Fisher Scientific) was used to amplify 100 ng of template DNA over 35 repeated cycles, consisting of 1 min of denaturation at 94 °C, 1 min of annealing at 60 °C, and 1 min of elongation at 72 °C.

VP7-HA amplicon was restriction digested with BamHI, and after treatment with T4 DNA polymerase to obtain a blunt-end fragment, it was cut with XbaI, and subcloned in pINT2EGFP [43], cut with NheI/SmaI (NheI and XbaI have compatible ends) to obtain pINT2-(TK-CMV-VP7-HA-TK).

### Transient transfection

HEK293T cells were seeded into 25 cm^2^ flasks (1 × 10^6^ cells/flask) and transfected with pINT2-(TK-CMV-VP7-HA-TK), or pEGFP-C1 (mock control, Clontech) using polyethyleneimine (PEI) transfection reagent (Polysciences, Inc.). DNA was mixed with PEI in a ratio 1:2.5 DNA:PEI in 500 μL of serum-free Dulbecco’s modified essential medium (DMEM) with high glucose (Euroclone) and incubated 15 min at room temperature, 4× volumes of serum-free medium were added, and the transfection solution was transferred onto the cells monolayer for 6 h at 37 °C with 5% CO_2_, in a humidified incubator.

### Immunoblotting

Western immunoblotting analysis was performed on protein cell extracts from HEK293T transfected with pINT2-(TK-CMV-VP7-HA-TK) or pEGFP-C1 (Clontech). BEK cells were also infected with 0.5 MOI of BoHV-4-A-CMV-VP7-HA-ΔTK or left uninfected and analyzed by western blotting. Membranes were probed with an anti-HA tag mouse monoclonal antibody (G036, Abm Inc., New York, NY, USA), diluted at 1:10 000, and horseradish-peroxidase (HRP)-labeled anti-mouse immunoglobulin (A9044, Sigma-Aldrick, (Merck), diluted at 1:15 000. The signals were visualized using enhanced chemiluminescence (Clarity Max Western ECL substrate, Bio-Rad, Hercules, CA, USA).

### Bacterial artificial chromosome (BAC) recombineering

BAC recombineering was carried out as previously described [40], in SW102 *Escherichia coli*, containing pBAC-BoHV-4-A-TK-KanaGalK-TK, retargeting the XhoI-linearized pINT2-(TK-CMV-VP7-HA-TK) into the BoHV-4 TK locus, containing the Kana/GalK selector cassette to obtain pBoHV-4-A-CMV-VP7-HA-ΔTK. BAC-DNA was purified and analyzed through HindIII restriction enzyme digestion and 1% agarose gel electrophoresis. Detailed protocols for recombineering can also be found at the recombineering website [63].

### Cell culture electroporation and recombinant virus reconstitution, production, and titration

BEK or BEKcre cells were maintained as a monolayer with cEMEM + 10% FBS. BAC-DNA (~5 µg) was electroporated in 600 μL DMEM (Euroclone) without serum (Bio-Rad Gene pulser Xcell, 270 V, 1500 µF, 4 mm gap cuvettes) into BEK and BEKcre cells. Cells were grown until the appearance of cytopathic effect (CPE). The virus was then harvested by freezing, thawing the cells three times, and the supernatant was titrated in BEK cells. The recombinant BoHV-4 was propagated by infecting confluent monolayers of BEK cells at a multiplicity of infection (MOI) of 0.5 of the 50% tissue culture infectious dose (TCID_50_). When the majority of the cell monolayer displayed the CPE (~48–72 hpi), the virus was harvested by freezing and thawing cells three times and pelleting the virions through a 30% sucrose cushion. Virus pellets were then resuspended in cold DMEM without FBS. The 50% tissue culture infectious dose (TCID_50_) was determined on BEK cells by limiting dilution.

### Virus growth curves

BEK cells were infected with BoHV-4-A and BoHV-4-A-CMV-VP7-HA-ΔTK at a MOI of 0.1 and incubated at 37 °C for 3 h. Infected cells were then washed with serum-free cEMEM and finally overlaid with cEMEM + 10% FBS. The supernatants were harvested daily, and the amount of infectious virus was determined by limiting dilution on BEK cells. Viral titer at each time point is reported as the average of triplicate measurements ± standard errors.

### Immunofluorescence (IF) staining and cytofluorimetric analyses of BoHV-4-A-CMV-VP7-HA-ΔTK infected cells

Sub-confluent monolayers of BEK cells were infected with 0.1 MOI of BoHV-4-A-CMV-VP7-HA-ΔTK or left uninfected. A total of 24 h after the infection, the cells were fixed with 4% paraformaldehyde for 10 min, washed three times with phosphate buffer saline (PBS) and then blocked and permeabilized with PBS containing 0.1% Triton-X 100, 1% bovine serum albumin (BSA), and 10% FBS for 1 h. Subsequently, the cells were incubated overnight at 4 °C with anti-HA mouse monoclonal antibody (G036, Abm Inc., Vancouver, BC, Canada) diluted at 1:1000 in PBS containing 1% BSA. After the removal of the antibody, the cells were washed extensively with PBS and incubated with the secondary antibody Alexa 488-conjugated goat anti-mouse IgG (A11029, Life Technologies) diluted at 1:500 1 h at room temperature. Cells were observed using inverted fluorescence microscopy (Zeiss-Axiovert-S100, Zeiss, Oberkochen, Germany), and images were acquired with a digital camera (Zeiss-Axiocam-MRC, Zeiss, Oberkochen, Germany).

The transduction efficiency of BoHV-4-A-CMV-VP7-HA-ΔTK in BEK cells was also assessed by flow cytometric analysis with a FACS Canto II (BD Biosciences). Briefly, infected BEK cells were detached with trypsin 48 hpi, washed, resuspended in cold PBS, and counted. One million cells were then fixed, permeabilized, and stained as described for the immunofluorescent staining protocol. The percentage of positive cells was determined by recording 50 000 events per sample using a gated strategy for Alexa-Fluor 488 signals based on the background signal from the cells probed with the mouse IgG3 isotype control (Invitrogen, 11-4742-42) at the same concentration as the anti-HA primary antibody (1 µg/test) and with the secondary antibody (1 µg/test). Data acquisition and analysis were carried out with Diva 9.02 software (BD Bioscience).

### In vivo mice model vaccinations

Female (7–8-week-old) IFNAR^−/−^ mice [44] on a C57BL/6 genetic background were housed in groups of five mice per cage (834 cm^2^ of floor area and 19 cm of height) in the Animal Facilities of Centro de Investigación en Sanidad Animal (CISA)–Instituto Nacional de Investigación y Tecnología Agraria y Alimentaria (INIA). The bedding was provided with a minimum of 2 cm of depth. Mice were immunized intraperitoneally (ip) twice (at 2-week intervals) with 100 μL containing 10^6^ TCID_50_ of BoHV-4-A-CMV-IgK-VP2gDtm or BoHV-4-A-CMV-VP7-HA-ΔTK; 200 μL of both BoHV-4 viral vectors (10^6^ TCID_50_/100 μL for each construct); or 100 μL (~10^6^ TCID_50_) of BoHV-4A-ΔTK, as control. To assess vaccination efficacy, mice were challenged intraperitoneally with 10^3^ PFUs of BTV-8 two weeks after the last immunization [35]. The power calculation was used to determine group size for these experiments [45]. All groups analyzed for vaccine efficacy contained ten mice. Mice were bled at day (D) 0, D15 (pre-boost), and 7 days post-boost. Subsequently, serum was obtained by centrifugation for 5 min at 10 000 × *g* and stored at −80 °C until use. Five mice per group were sacrificed on day 7 post-boost to analyze T-cell responses using splenocytes [42], and 15 days after the booster immunization, the five animals remaining per group were challenged with BTV-8. The challenged mice were monitored daily for disease signs starting at 24-hpi and up to day 14 post-challenge (PC) and were bled at days 3, 5, 7, 9, 10, and 15 PC to determine viremia in blood. Mice were euthanized to stop pain or distress according to the humane endpoints in our animal protocols. The euthanasia method used was an overdose of inhalant anesthesia (isofluorane) and cervical dislocation.

### Enzyme-linked immunoadsorption assay (ELISA) for the detection of immunoglobulin G (IgG) against BTV-8

ELISA plates (Maxisorp, Thermo Fisher Scientific,Waltham,MA, USA) were coated with 10^4^ PFU/well of the purified virus (BTV-8), incubating overnight at 4 °C. The next day, after a blockade with PBS + 5% milk, different dilutions of inactivated serum (56 °C, 30 min) of the inoculated mice were added, diluted in PBS + 1% milk, and incubated for 1 h. Total IgGs anti-BTV-8 were detected using an HRP-conjugated anti-mouse IgG secondary antibody (0.1 µg/mL) (Biorad). After several washes, the plates were developed with 3,3′,5,5′-tetramethylbenzidine (TMB) (Thermo Fisher Scientific, Waltham, MA, USA). The reaction was quenched with 3N sulfuric acid, and the absorbance at 450 nm was measured on a FLUOstar Omega ELISA plate reader (BMG Labtech, Ortenberg, Germany). IgG titer are expressed as the reciprocal value using a linear regression of the serum dilutions for which OD_450_ nm reading in immune serum dilution reaches two times that of the OD_450_ nm reading in the pre-immune serum of the same animal as previously described [46].

### Viral RNA extraction and quantification by quantitative PCR (qPCR)

Real-time qPCR (RT-qPCR) experiments were conducted and reported in accordance with the Minimum Information for Publication of Quantitative Real-Time PCR Experiments (MIQE) guidelines. Total RNA was extracted from 50 μL of total blood using the trizol reagent solution (Invitrogen) following the manufacturer’s instructions, including on-column DNase treatment. RNA concentration and purity were determined by spectrophotometry (A260/280).

We used 50 ng/μL of total blood RNA to perform a one-step RT-qPCR specific BTV segment 5 amplification with Luna^®^ Universal Kit (New England Biolabs). Primers forward 5′-GTTGAATTGGCAAAGGAGGCAATG-′3 and Reverse 5′-GGGATGATGGATGAGGCCGTG-3′ in a final volume of 20 μL were used to amplify 165pb fragments of segment 5 of BTV.

The viral RNA quantification was performed on a MX3005P thermocycler (Agilent) using SYBR Green/probe-based chemistry. The cycling conditions were as follows: an initial denaturation step at 95 °C for 7 min to ensure complete strand separation, followed by reverse transcription at 57 °C for 10 min and a second denaturation at 95 °C for 1 min. Amplification was carried out for 40 cycles consisting of denaturation at 95 °C for 10 s and extension at 70 °C for 30 s. A melting curve analysis was subsequently performed with the following steps: 95 °C for 1 min, 70 °C for 30 s, and 95 °C for 30 s. Melt curve analysis confirmed the amplification of a single specific product. No-template controls and minus-RT controls were included in all runs.

### Bluetongue virus seroneutralization assays

The sera obtained from the vaccinated animals at days 0 and 14 post-boost were inactivated for 30 min at 56 °C. Serial dilutions (1/20, 1/40, 1/80, and 1/160) of inactivated sera were incubated with 100 plaque forming units (PFU) of virus (BTV-8) in 96-well plates for 1 h at 37 °C. Subsequently, Vero cells (2 × 10^4^ cells/well) were added and incubated at 37 °C, with a 5% CO_2_ atmosphere and with a relative humidity of 98%. After 4–5 days, the presence or absence of lysis were determined by fixing and staining with crystal violet in 2% paraformaldehyde. Serum neutralization titers are represented as the dilution at which 50% of the monolayer is lysed.

### Enzyme-linked immunoadsorption assay (ELISPOT) for the detection of interferon gamma (IFN-γ)

Splenocytes were obtained as described previously [47, 48] and cultured in Roswell Park Memorial Institute medium (RPMI) supplemented with 10% FBS (Merck), 4 mM L-glutamine, 10 mM 4-(2-hydroxyethyl)-1-piperazineethanesulfonic acid (HEPES), 1% 100 × nonessential amino acids, 1 mM sodium pyruvate, 100 U/mL penicillin/100 μg/mL streptomycin, and 50 nM β-mercaptoethanol (all from Gibco, Thermofisher). Splenocytes were seeded at a density of 2 × 10^5^ cells per well in a MSIPS4510 plate (Millipore) coated with capture antibody (5 µg/mL) from BD™ ELISPOT Mouse IFN-γ ELISPOT Pair (BD Biosciences) in the presence of purified and inactivated BTV-8 (equivalent to 1 × 10^4^ PFU prior to inactivation) or a pool of VP7 protein peptides [10 µg/mL; VP7(139) GRWFMRAAQAVTAVV; VP7(324) RPEFAIHGVNPMPGP; VP7(72) AAGINVGPI; VP7(80) ISPDYTQHM; VP7(283) TAILNRTTL; VP7(327) FAIHGVNPM described in [47, 49]] as stimulus. As a negative control we used DMEM, and as a positive control concanavalin A (1.25 µg/mL). After 24 h incubation, cells were discarded and membranes incubated with biotin-labelled detection antibody (2 µg/mL) (BD™ ELISPOT Mouse IFN-γ ELISPOT Pair; BD Biosciences). Membranes were then were incubated for 1 h with alkaline phosphatase-conjugated streptavidin (ExtrAvidin-AP, from Sigma) diluted 1:10,000 in PBS + 0.5% FBS. After several washes, the membranes were revealed using Sigma FAST BCIP/NBT (Sigma). The result was read in an ELISPOT reader (AID iSpot reader).

### Intracellular cytokine (ICS) staining and flow cytometry

Splenocytes from vaccinated mice were cultured at a density of 10^6^ cells per well in the presence of inactivated virus (iBTV) as described by Rojas et al. [42]. As a negative control RPMI was used, and RPMI medium supplemented with 50 ng/mL of phorbol 12-myristate 14-acetate (PMA) + 1 μg/mL of ionomycin was used as a positive control. Subsequently, a mixture of brefeldin-A (5 µg/mL) + monensin (2 µM) + anti-CD107a-PE (1 µg/mL) (all from Biolegend) was added and incubated for 5 h, in an atmosphere with 5% CO_2_ and 37 °C. This allows blocking of cytokine secretion and allows labeling with CD107a to determine degranulation. After stimulation, cells were stained for 20 min on ice with a viability marker of live–dead cells (1:1000 dilution of LIVE/DEAD Near-IR, Thermofisher) and then after one wash with different antibodies (all used at 2 µg/mL): anti-CD3-AF700, mouse anti-CD4-BV510, and anti-CD8a-AF488 (all from Biolegend) for 20 min on ice. The cells were fixed and permeabilized using the BD Cytofix/Cytoperm kit (BD) as described in the manufacturer’s protocol. Subsequently, they were labeled with different intracellular antibodies (all used at 2 µg/mL): anti-IFN-γ- AF647, mouse anti-TNF-α-BV421, and anti-IL2-BV786 (All from Biolegend) for 25 min on ice, and the fluorescence was detected with a FACSCelestaSorp flow cytometer (Becton Dickinson). The data were analyzed with the FlowJo software (TreeStarInc). Gating strategy and staining examples are presented in Additional file 1.

### Statistical analysis

Normality was assessed with the Shapiro–Wilk test. Statistical data analysis was performed with Graphpad Prism 9 on the basis of the dataset structure (GraphPad Software, Inc., USA). Homogeneity of variance was tested using a Brown–Forsythe test. The data were evaluated using different analyses: using an unpaired two-tailed Student’s *t*-test with a correction by the test of Welch; a one-way analysis of variance (ANOVA) with a correction using the Newman–Keuls test; and a two-way multiple comparisons ANOVA with a correction using the Dunnet’s test or Tukey’s test. A confidence interval of 0.05 was used for all analyses.

## Results

### Generation of recombinant bovine herpes virus expressing BTV-8-VP7 (BoHV-4-A-CMV-VP7-HA-ΔTK) protein

**Figure 1:**
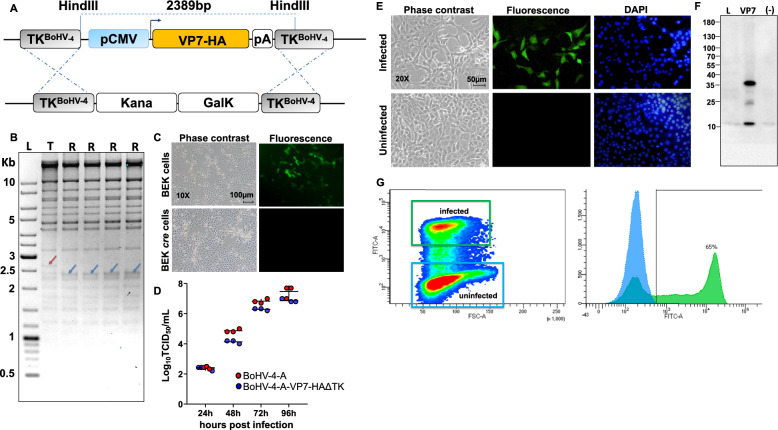
**Generation and characterization of BoHV-4-A-VP7-HA-ΔTK**. **A** Diagram describing the retargeting event obtained by heat-inducible homologous recombination in SW102 containing pBAC-BoHV-4-A-TK-KanaGalK-TK, where the Kana/GalK cassette was replaced with the CMV-VP7-HA expression cassettes flanked by BoHV-4 TK sequences, located in pINT2 shuttle plasmid vector (pINT2-(TK-CMV-VP7-HA-TK). **B** Representative 2-deoxy-galactose resistant colonies [pBoHV-4-A-VP7-HAΔTK; R (Retargeted)] tested by HindIII in agar gel electrophoresis and compared with the parental pBoHV-4-A-Kana/GalKΔTK [T (Targeted)]. The ~2650 bp band (blue arrow), corresponding to the un-retargeted pBAC-BoHV-4-A-TK-KanaGalK-TK control, has been replaced by a 2389 bp band in BoHV-4-A-VP7-HA-ΔTK (red arrow) and is not distinguishable because of overlapping with other bands of the same size. Four lanes (R, R, R, and R) correspond to the same clone analyzed at the second, fourth, sixth, and eighth passages, respectively, thereby demonstrating its stability over time. **C** Representative images of phase contrast and fluorescent fields of plaques formed by viable reconstituted recombinant BoHV-4-VP7-HAΔTK after the corresponding BAC DNA was electroporated into BEK cells or in BEK cells expressing *cre* recombinase (×10). **D** Replication kinetics of BoHV-4-A-VP7-HA-ΔTK in BEK cells compared with those of the parental BoHV-4-A isolate. Data are presented as means ± standard errors from three independent replicates for each time point (*P* > 0.05, Student’s *t*-test). **E** Immunofluorescent staining of BoHV-4-A-VP7-HAΔTK-infected BEK cells and the uninfected control. The immunostaining was performed with an anti-HA mAb, counterstained with 4′,6-diamidino-2-phenylindole (DAPI). **F** Western immunoblotting of cells infected with BoHV-4-A-VP7-HA-ΔTK (VP7), the negative control (−) with protein extract from BoHV-4-A infected cells. The lanes were loaded with 20 μg of protein extract. **G** Cell surface expression of VP7 by 0.1 MOI BoHV-4-A-VP7-HA-ΔTK infected BEK cells at 48 h post-infection as measured by flow cytometry. Overlayed density and histogram plots distinguishing infected cells expressing VP7 (green square) from the uninfected control (blue square), where ~65% of cells were positive.

VP7 of BTV-8, the primary inner capsid protein and group-specific antigen [50], contains immunodominant, serotype-crossreactive T-cell epitopes. However, it does not produce neutralizing antibodies against intact virus particles. The induction of a crossreactive, cell-mediated immune response may also provide crossreactive protection within the same animal. This has been demonstrated through adoptive transfer techniques, suggesting that cell-mediated immunity could play a crucial role in developing a protective immune response to BTV without neutralizing antibodies. Based on these concepts, the study aimed to evaluate the immunogenicity and protective potential of VP7 when expressed by the BoHV-4 vector. To this end, a recombinant BoHV-4 vector expressing VP7 was developed. Initially, a suitable expression cassette capable of efficiently driving the expression of the VP7 ORF was constructed. The VP7 open reading frame (ORF) sequence was provided, with a hemagglutinin (HA) tag added to its carboxy terminal to facilitate tracking of the expression. VP7-HA was subcloned into a BoHV-4 pINT2 shuttle vector, which contains two BoHV-4 TK gene homologous sequences, a CMV promoter, and a bovine growth hormone polyadenylation signal, to generate pINT2-(TK-CMV-VP7-HA-TK). HEK 293 T cells transfected with pINT2-(TK-CMV-VP7-HA-TK) successfully expressed VP7-HA (Additional file 2). The upper band represents the full-length VP7 protein, whereas the lower band corresponds to a proteolytic fragment. Because the extract was prepared from infected cells, viral replication typically disrupts normal cellular compartmentalization, thereby enhancing proteolytic activity. This increase in proteolysis can facilitate antigen processing and presentation, a beneficial feature commonly associated with viral-vector-based systems. The BoHV-4-A-CMV-VP7-HA-ΔTK, which carries the optimized CMV-VP7-HA expression cassette, was created through heat-inducible homologous recombination in an SW102 *E. coli* strain containing pBAC-BoHV-4-A-KanaGalK-ΔTK (Figure 1A). The authenticity of the pBAC-BoHV-4-A-CMV-VP7-HA-ΔTK viral genome was initially verified using HindIII restriction enzyme analysis (Figure 1B). Clonal stability was ensured by culturing the positive clone over 20 passages (data not shown). Infectious viral particles of BoHV-4-A-CMV-VP7-HA-ΔTK were then produced via electroporation of BEK or BEKcre cells. BEKcre cells demonstrated the removal of the BAC/GFP cassette from the recombinant viral genome, indicated by the absence of green plaques (Figure 1C). Furthermore, BoHV-4-A-CMV-VP7-HA-ΔTK exhibited no significant replication defects compared with the BoHV-4-A parental strain (Figure 1D) and expressed the VP7-HA protein, as evidenced by IF staining (Figure 1E), western blotting (Figure 1F), and flow-cytometry (Figure 1G).

### Vaccination with BoHV-4-A-CMV-VP7-HA-ΔTK and BoHV-4-A-CMV-IgK-VP2gDtm induces systemic BTV-specific antibodies but different levels of neutralizing antibodies

**Figure 2:**
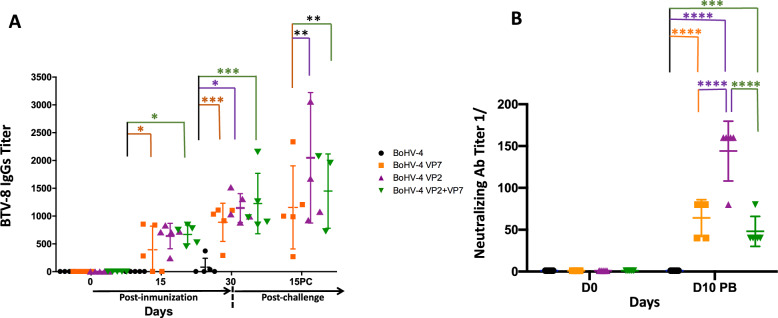
**Serum IgGs and neutralizing antibodies response in mice following immunization with recombinant BoHV-4s.** **A** Antibody responses elicited against BTV-8 virus assessed by ELISA. Data are expressed as individual values obtained for each animal, which are the reciprocal of the highest dilution of serum that gives an ODA450 of twice the value obtained with the animal pre-immune serum. **B** Neutralizing antibody response before challenge of vaccinated and control mice. *p < 0.05; ^**^p < 0.01; and ^***^p < 0.001 in two-way multiple-comparisons ANOVA with Dunnet’s or Tukey’s post-test.

BTV-specific antibodies were quantitated by ELISA in serum of mice vaccinated with the different recombinant BoHV-4-based vaccines. Anti-BTV-8 IgG levels were detectable in all vaccinated animals by day 15 following the initial immunization (Figure 2A). The mean values of the three groups almost doubled after the booster dose and show a slight increase after viral challenge while mice treated with BoHV-4A-ΔTK control vector remained seronegative prior to the challenge. Interestingly, before the challenge, total IgGs against BTV-8 tended to be higher in the VP2 + VP7 vaccinated group when compared with the monoantigenic immunized groups. After challenge, the highest IgG values were reached in the VP2 vaccinated group with statistically significant differences versus the two other vaccinated groups. Overall, the VP7 immunized group presented the lowest IgG values.

Neutralization assays were also carried out to determine the level of specific neutralizing antibodies to BTV-8 in sera from animals by day 10 post-booster. BTV-specific neutralizing antibodies were detected in the three vaccinated groups, but titers were statistically superior in the BoHV-4-A-CMV-IgK-VP2gDtm vaccinated group. The bi-vaccinated group showed the lowest neutralization activity (Figure 2B). Taken together, these data suggest that all recombinant antigens were immunogenic, inducing the production of BTV-8-specific antibodies but the best inducer of neutralizing antibodies against BTV-8 is the strategy in which VP2 is administrated alone in a recombinant BoHV-4A-ΔTK-based vector.

### Recombinant bovine herpes-4 viruses induce a specific T cell response

**Figure 3:**
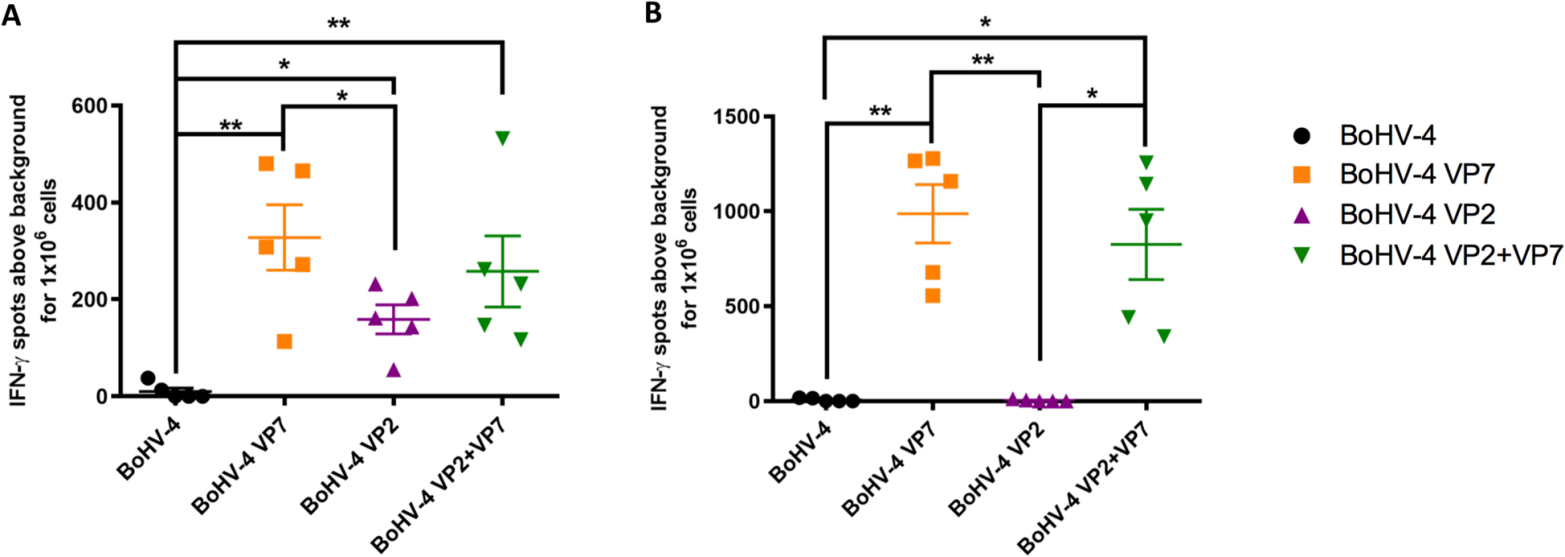
**BoHV-4 vaccination induces IFN-γ T-cell responses.** Splenocytes were seeded in anti-IFN-γ-coated ELISPOT plates and stimulated overnight with **A** iBTV or **B** a VP7 immunogenic peptide pool. Splenocytes were discarded, and ELISPOT plates revealed for IFN-γ production. ^*^p < 0.05; ^**^p < 0.01; and ^***^p < 0.001. One-way ANOVA with Fisher’s least significant difference (LSD) post-test.

While humoral immunity is traditionally considered the primary goal of vaccination and a key factor in protection against pathogens, it is now well established that cellular immune responses also play a critical role in protection against BTV [17, 38]. Therefore, we analyzed the cellular response induced by the potential BoHV-4-vaccines used in this study. Firstly, the IFN-γ response of iBTV-stimulated splenocytes was determined 7 days after booster immunization. All immunized mice showed BTV-specific cellular responses measured by IFN-γ ELISPOT assay (Figure 3A). The BoHV-4A-BTV-8-VP7-ΔTK vaccinated group showed the highest level of IFN-γ-secreting cells, in response to both stimuli, iBTV or a pool of specific immunogenic VP7 peptides [41] (Figure 3B). As expected, VP2 vaccinated group showed lower IFN-γ values against iBTV when compared with the VP7-vaccinated group (Figure 3A) and no response against the VP7 peptides pool (Figure 3B). Including VP7 in the vaccination formulation alongside VP2 tended to increase the IFN-γ response compared with vaccination with VP2 alone, although this did not reach statistical significance. No IFN-γ production was detected in the nonvaccinated control group.

**Figure 4:**
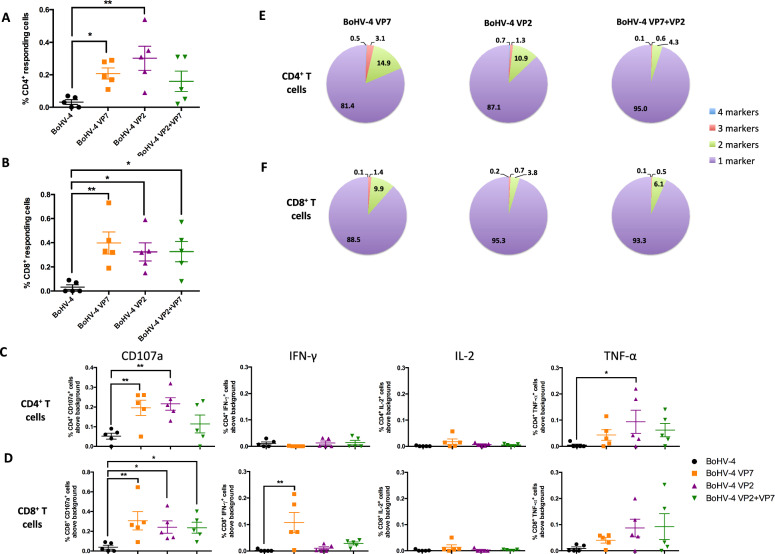
**BoHV-4 vaccination induces BTV-specific polyfunctional T cells.** Splenocytes were stimulated with iBTV, and expression of CD107a, IFN-γ, IL-2, and TNF-α was assessed in CD4^+^ and CD8^+^ T cells by intracellular cytokine staining and flow cytometry analysis. **A and B** Total percentage of **A** CD4^+^ or **B** CD8^+^ T cells responding to iBTV stimulation (i.e., expressing at least one stimulation marker). **C and D** Expression of CD107a, IFN-γ, IL-2, and TNF-α in **C** CD4^+^ T cells and **D** CD8^+^ T cells. ^*^p < 0.05 and ^**^p < 0.01. One way ANOVA with Fisher’s least significant difference (LSD) post-test. **E and F** Percentage of simultaneous expression of one, two, three, or four stimulation markers in **E** CD4^+^ T cells and **F** CD8^+^ T cells in response to iBTV for each vaccine regime.

To further characterize the cellular immune response induced by vaccination, we evaluated by flow cytometry the expression of four key functional markers: IFN-γ, TNF-α, IL-2, and CD107a on CD4^+^ and CD8^+^ T cells stimulated with iBTV (Figure 4; Additional file 1). Results showed that all vaccination regimes induced BTV-specific CD4^+^ and CD8^+^ T cells expressing at least one of these markers (Figure 4A, B). Notably, CD107a expression increased after vaccination, indicating the cytotoxic potential of both T-cell subsets (Figure 4C, D). IFN-γ production was detected in CD8^+^ T cells from the VP7-vaccinated group, whereas VP2 and VP2 + VP7 groups showed stronger TNF-α responses in both CD4^+^ and CD8^+^ T cells (Figure 4C, D). Although most BTV-specific T cells expressed a single functional marker, some polyfunctional T cells, those positive for two or more markers, were detected predominantly in the VP7 vaccinated group (Figure 4E, F). The presence of polyfunctional T cells is a feature of effective and durable cellular immunity, as these cells are associated with enhanced antiviral activity and long-term protection. These findings highlight the immunogenic potential of VP7 in vaccine formulations against BTV, especially in driving robust and potentially multifunctional T-cell responses.

### Immunization with bovine BoHV-4-A-CMV-IgK-VP2gDtm and/or BoHV-4-A-CMV-VP7-HA-ΔTK induces protection against lethal challenge with BTV-8 in IFNAR^−/−^ mice

**Figure 5:**
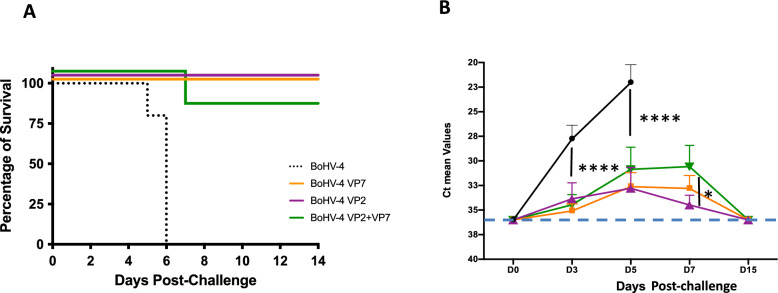
**Protection in BoHV-4-vaccinated mice after BTV-8 challenge**. **A **Mice survival. IFNAR^−/−^ mice were vaccinated with an empty BoHV-4 (BoHV-4-A-ΔTK) as control, a BoHV-4 expressing VP7, (BoHV-4 VP7; BoHV-4-A-CMV-VP7-HA-ΔTK) or a BoHV-4 expressing VP2 (BoHV-4 VP2; BoHV-4-A-CMV-IgK-VP2gDtm) or were co-vaccinated with both BoHV-4 expressing either VP7 and VP2 (BoHV-4 VP2 + VP7; BoHV-4-A-CMV-VP7-HA-ΔTK + BoHV-4-A-CMV-IgK-VP2gDtm). Animals were challenged with 10^3^ PFU BTV-8 intraperitoneally and monitored daily for the appearance of clinical signs. Animals were sacrificed when they reached the defined humane endpoint. Percentage of survival in each group is represented. Survival plots were offset (all groups start at 100%) to improve figure readability. **B **RT-qPCR. Blood was collected from the control (BoHV-4 Control) and vaccinated (BoHV-4 BTV-8-VP7, BoHV-4 BTV-8-VP2, and BoHV-4 BTV-8-VP7 + VP2) mice at indicated times post-BTV-8 challenge. Total RNA was extracted and RT-qPCR performed. Results are expressed as Ct. The cut-off value is indicated with a dotted line (Ct = 37). ^*^p < 0.05 and ^****^p < 0.0001. Two-way ANOVA with Tukey’s multiple comparison test.

The IFNAR^−/−^ mice model, a valid surrogate model to study the effectiveness of BTV vaccines [4, 35, 51–54]*,* was used to determine protection against BTV lethal challenge by the recombinant BoHV-4 vaccines. To this end, four groups of mice were inoculated with either BoHV-4-A-CMV-VP7-HA-ΔTK, BoHV-4-A-CMV-IgK-VP2gDtm, or the empty control vector. Monoantigenic BoHV-4 vectors expressing BTV-8 VP2 or BTV-8 VP7 proteins, respectively, and administered individually, conferred total protection in IFNAR^−/−^ in mice. By contrast, vaccination with the empty vector resulted in all mice succumbing to the disease (Figure 5A). An 80% of protection was observed in the group vaccinated with two antigens (green line in Figure 5A). Regarding viral RNA load in blood (Figure 5B), control animals showed decreasing cycle threshold (Ct) values from 28–25 at day 3 to 24–20 at day 7, which coincided with their death. In the BoHV-4-A-CMV-IgK-VP2gDtm vaccinated group, two out five animals showed Ct values above 33 throughout, two others had Ct values below 35, and only one mouse reached Ct values of 24 at D5 PC, which subsequently increased to 30 at D7 and became undetectable by D15 PC. In the BoHV-4-A-CMV-VP7-HA-ΔTK vaccinated group, two animals are fully protected, showing no detectable viral RNA at any time. Among the remaining three, peak Ct values of 29–30 were observed at day 5 or 7, with viral RNA becoming undetectable by day 15. The group vaccinated with both antigens exhibited a delay in the viral RNA peak with significantly lower Ct values compared with controls. Ct values in this group ranged between 29 and 31 at days 5 and 7 and became undetectable at day 15, except for in one animal that reached a Ct of 22 on days 5 and 7 and died at D7 PC (Figure 5B). Thus, the protection detected correlated with a statistically significant decrease of the viral RNA determined in blood by RT-qPCR when comparing vaccinated animals with controls at D3 and D5 PC. Within the low viral RNA detected in all vaccinated animal during the experiment, the values in the VP2 + VP7 vaccinated group were above the other vaccinated groups, and this was statistically significant at day 7 PC. Importantly, viral RNA became undetectable at D15 after challenge in all vaccinated groups (Figure 5B). These data indicate that all mice immunized with the BoHV-4 vectors expressing BTV-VP7, BTV-VP2, or both were able to elicit both humoral and cellular immune responses against BTV, which was potent enough to confer protection (Figure 5).

## Discussion

Although BTV vaccines are available, they are based on virus inactivation and face disadvantages, failing to fulfill the principle of differentiating infected from vaccinated animals (DIVA) or providing limited cross-protection among different BTV serotypes [12, 14]. The recent severe BTV serotype 3 outbreaks that occurred in the Netherlands in 2023 and spread across Europe reaching Scandinavia and Greece in 2024, or the recently confirmed cases of serotype 12 in the Netherlands and the UK [9, 10, 55] illustrate the threat that the emergence of new BTV serotypes poses to the ruminant industry. Control of the disease is further complicated by the difficulty in achieving total protection with the current vaccines, as exemplified with inactivated BTV-3 vaccines, which underlines the necessity to obtain more potent DIVA BTV vaccines.

BoHV-4 shares the ability as a BTV antigen delivery vehicle with other recombinant-virus-based systems, such as adenovirus [17], poxvirus [18, 19], herpesvirus [56], phlebovirus [57], and rhabdovirus. The BTV antigens expressed in these different vectors, vary from capsid proteins such as VP2 [39, 58], VP5 [19], or VP7 [59], to nonstructural proteins such as NS1 [23] or NS3 [17, 60] or different combinations of these. The initial target antigens assayed in recombinant vaccine systems were those expected to elicit a strong humoral response with high neutralizing antibodies titers, such as VP2—primarily—and VP5. However, it has been shown that the ability to induce a T-cell response is also a key correlate of protection, particularly for cross-protection [4]. Therefore, the focus has recently shifted toward the inclusion in vaccine formulation of viral proteins conserved among serotypes, such as NS1, NS2, NS3, or VP7.

Thus, this study provides an in-depth re-evaluation of the vaccine efficacy and immunogenicity of modified live BoHV-4 vectors expressing the most antigenic and immunodominant capsid proteins from BTV, the glycoprotein VP2 (BoHV-4-A-CMV-IgK-VP2gDtm), previously used in Capocefalo et al. [39], which unveiled novel immune response aspects and protection against virulent challenge. Moreover, a new recombinant BoHV-4A-ΔTK expressing the conserved BTV-8 VP7 protein (BoHV-4-A-CMV-VP7-HA-ΔTK) was developed and included in the present immunologic and protective study, alone or in combination with the BoHV-4-A-CMV-IgK-VP2gDtm [39].

The IFNAR^−/−^ mouse model, employed to analyze potential vaccines against different pathogens due to their capability to reproduce different maladies signs and outcome such as BT [18, 35], was used to evaluate the new vaccine prototype based on the BoHV-4A-ΔTK platform that delivers and expresses VP7. Direct assessment of vaccine efficacy in large animals is resource-intensive and challenging, particularly in high-containment facilities, whereas murine infection models provide relevant, rapid, and cost-effective data that can reliably predict vaccine immunogenicity in the natural host [37, 38]. The absence of disease in the BoHV-4A-ΔTK inoculated IFNAR^−/−^ mice was already established in a previous study [39], which we confirmed here, as no adverse effects were detected in vaccinated mice. This is an important issue to address with recombinant viral vaccines due to the high viral disease susceptibility that this mouse model possesses. The IFNAR^−/−^ mouse model is consequently suitable for vaccination studies using BoHV-4 vector vaccines. Moreover, BoHV-4A-ΔTK-based vector have been shown to behave like a replicating incompetent viral vector in both wild-type and immunocompromised mice, with complete absence of pathogenicity [43]. In the present work we inoculated 40 mice with different BoHV-4 constructs, and no animal developed clinical signs, which confirmed the safety of this vector in IFNAR ^(−/−)^ mice.

Although higher IgG and neutralizing antibody titers were observed in the sera of mice vaccinated with BoHV-4-A-CMV-IgK-VP2gDtm (Figure 2), all recombinant vaccines were immunogenic, inducing the production of BTV-8-specific antibodies from the first inoculation, with levels increasing after the booster (Figure 1A). In contrast, previous work in a similar murine model only elicited low neutralizing antibody titers [39]. This could be due to the different genetic background of the IFNAR ^(−/−)^ mice used in these studies. Interestingly, we detected low level of neutralization in the BoHV-4-A-CMV-VP7-HA-ΔTK vaccinated group. This has not been reported with BTV, but neutralization against proteins from the inner core of other *Sedoreoviridae* has been documented. Further work will be required to evaluate the capacity of BoHV-4 vector to elicit this type of response.

After challenge, all control animals succumbed by day 6 PC, in contrast to the complete survival of vaccinated mice, with the exception of a single death in the bi-antigenic vaccinated group (Figure 5A). This outcome differs significantly from the 5-day delay in mortality, except for one survivor, previously reported with BoHV-4-A-CMV-IgK-VP2gDtm-vaccinated and BTV-8-challenged mice [39]. Our survival data agree with the induction of neutralizing antibody titers in the IFNAR ^(−/−)^ C57BL/6 background with the BoHV-4-A-CMV-IgK-VP2gDtm vaccination. This supports the idea that the differences in survival observed between the present study and the previous one by Franceschi et al. [39] may be attributed to variations in vaccine response resulting from differences in the genetic background of the mouse strains used. Overall it appears that VP7 inclusion in the vaccine formulation could be beneficial, as it provided full protection in this murine model. This is in line with previous work that indicated that VP7 could represent a vaccine target for the control of BTV [4, 17].

A previous study evaluating the potential of the BoHV-4-A-CMV-IgK-VP2gDtm vaccine against BTV-8 did not assess the T-cell responses elicited in mice following immunization with the BoHV-4-based vector [39]. Our work demonstrates that both BoHV-4-A-CMV-IgK-VP2gDtm and BoHV-4-A-CMV-VP7-HA-ΔTK vaccines were capable of inducing BTV-specific CD4^+^ and CD8^+^ T-cell responses. Notably, only the groups immunized with the BoHV-4-A-CMV-VP7-HA-ΔTK vector developed measurable T-cell responses against VP7 immunogenic peptides, indicating the robust immunogenicity of this capsid protein when delivered through the BoHV-4 vector. It significantly increased IFN-γ secretion in response to both whole virus and VP7 peptides, and further enhanced T-cell responses when co-administered with BoHV-4-A-CMV-IgK-VP2gDtm (Figure 3A). In BoHV-4-A-CMV-VP7-HA-ΔTK vaccinated groups, a higher proportion of CD8^+^ T-cell responders was observed compared with CD4^+^ T cells, suggesting a Th1-biased immune response. Interestingly, although CD8^+^ T-cell activation was relatively consistent across all vaccinated groups, the proportion of CD4^+^ T-cell responders tended to be slightly higher in the VP2-only vaccinated group. This potential differential activation may reflect differences in antigen processing and presentation pathways associated with each antigen. Importantly, VP7 is a highly conserved inner capsid protein across different BTV serotypes [41], and T-cell epitopes within VP7 have been shown to be crossreactive among multiple serotypes [4]. The potent VP7-specific cellular immune response induced by BoHV-4-A-CMV-VP7-HA-ΔTK, comparable to that achieved by adenoviral vectors (human type 5 [17] or ChAdOx1 [23]), could therefore confer cross-serotype protection. This could complement the more serotype-restricted neutralizing antibody responses typically directed against the outer capsid protein VP2. The induction of VP7-specific CD8^+^ T cells may be particularly relevant for viral clearance and protection against heterologous BTV strains, given their potential to recognize infected cells across serotypes and mediate cytolytic activity. These findings support the inclusion of conserved antigens such as VP7 in vaccine strategies aimed at broadening the protection against diverse BTV serotypes.

Although this work was not performed in sheep, the translational value of murine models is supported by previous studies on peste des petits ruminants virus (PPRV). In that context, intraperitoneal immunization in mice with a BoHV-4–based vector (as in other studies using this vector [39]) expressing the PPRV hemagglutinin antigen elicited strong humoral and cellular immune responses [37], providing an early indication of its immunogenic potential. Importantly, these findings were later confirmed in the natural host, where intramuscular immunization of sheep with the same construct conferred protection against virulent challenge and prevented virus shedding [38]. While it should be noted that the route of administration can influence vaccine immunogenicity by affecting antigen distribution, uptake, and immune priming [61, 62]—with intraperitoneal delivery typically favoring systemic exposure and intramuscular or subcutaneous routes targeting different antigen-presenting cells—the concordance between murine and ovine data highlights the robustness of this platform. Indeed, the successful translation of immunogenicity data across species [37, 38], even with differences in the administration routes, suggests that BoHV-4-based vectors retain their capacity to induce protective immunity when administered intramuscularly in ruminants, reinforcing the relevance of the mouse model as an effective preclinical tool for vaccine evaluation.

Overall, the work presented here aligns with next-generation vaccine strategies—such as DIVA-compliant, universal, or multiserotype vaccines—against bluetongue, showing robust induction of humoral and cellular immune responses against BTV that lead to protection. Given the nature of the vector used, dual protection against bovine herpesviruses (type 1, for example) cannot be excluded and warrants further investigation. Based on these findings, the next step would be to evaluate vaccine efficacy in target species of the disease, such as sheep or calves. Expression of VP2 and VP7 in BoHV-4 vectors therefore represents a promising strategy that could help control the spread of BTV across new geographical areas.

## Supplementary Information

**Additional file 1. Gating strategy and representative examples of intracellular cytokine staining assays analyzed by flow cytometry.** Splenocytes from vaccinated mice (BoHV-4, BoHV-4 VP7, BoHV-4 VP2, and BoHV-4 VP2+VP7) were left unstimulated as control or were stimulated with inactivated BTV-8 (i-BTV). Gating strategy consisted in selecting the lymphocyte population by FSC-A versus SSC-A discrimination, excluding doublets (FSC-A versus FCS-H, followed by SSC-A versus SSC-H), selecting live cell events by discrimination with the viability marker live/dead near-infrared (L/D NIR), and gating the CD3^+^ CD4^+^ events for CD4^+^ T cells, or the CD3^+^ CD8^+^ events for CD8^+^ T cells, as shown. Degranulation (CD107a) and cytokine production (IFN-γ, IL-2, and TNF-α) in CD4^+^ T cells and CD8^+^ T cells were assessed in unstimulated (as control) and i-BTV-stimulated cultures. Representative dot-plots of i-BTV-stimulated cultures are shown for each parameter from BoHV-4, BoHV-4 VP7, BoHV-4 VP2, and BoHV-4 VP2 + VP7 vaccinated mice. Representative dot-plots of unstimulated cells from BoHV-4 VP7 responding mice (i.e., background production) are shown as control.

**Additional file 2. Staining of pINT2-(TK-CMV-VP7-HA-TK)-transfected HEK293 cells and stained with an anti-HA mAb antibody and analyzed by immunofluorescence and flow cytometry.**

## Data Availability

The datasets used and/or analyzed during the current study are available from the authors on reasonable request.
