# Supplementary figures and images for: Expression of bluetongue virus core proteins VP2 and VP7 by bovine herpesvirus-4 confers protection against virulent challenge in a mouse model
Source: Vet Res. 2026 May 28;57:97. doi: 10.1186/s13567-026-01771-5 (PMC13220488; doi:10.1186/s13567-026-01771-5)

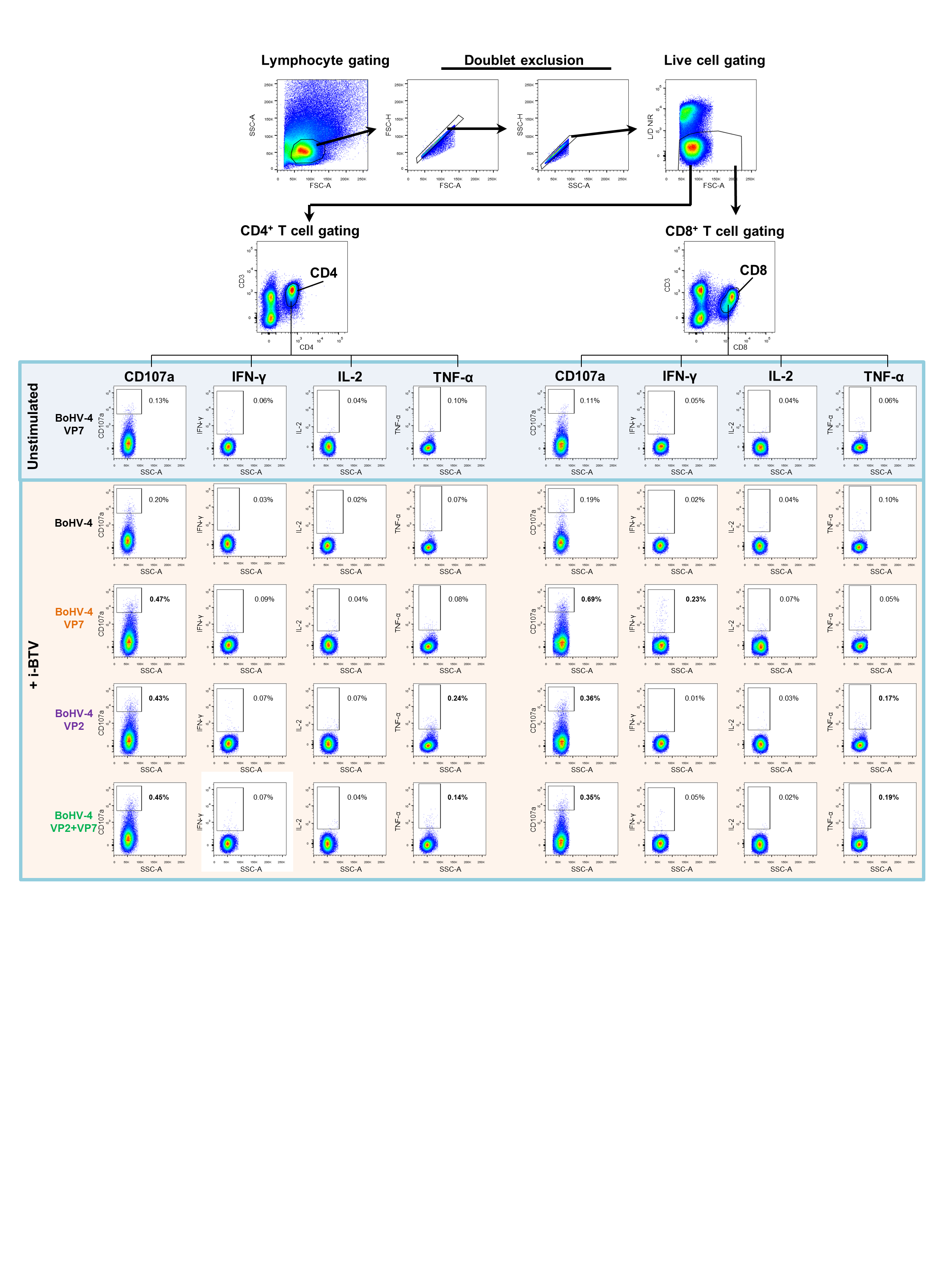

Supplement: Supplementary file 1 — Additional file 1. Gating strategy and representative examples of intracellular cytokine staining assays analyzed by flow cytometry. Splenocytes from vaccinated mice (BoHV-4, BoHV-4 VP7, BoHV-4 VP2, and BoHV-4 VP2+VP7) were left unstimulated as control or were stimulated with inactivated BTV-8 (i-BTV). Gating strategy consisted in selecting the lymphocyte population by FSC-A versus SSC-A discrimination, excluding doublets (FSC-A versus FCS-H, followed by SSC-A versus SSC-H), selecting live cell events by discrimination with the viability marker live/dead near-infrared (L/D NIR), and gating the CD3+ CD4+ events for CD4+ T cells, or the CD3+ CD8+ events for CD8+ T cells, as shown. Degranulation (CD107a) and cytokine production (IFN-γ, IL-2, and TNF-α) in CD4+ T cells and CD8+ T cells were assessed in unstimulated (as control) and i-BTV-stimulated cultures. Representative dot-plots of i-BTV-stimulated cultures are shown for each parameter from BoHV-4, BoHV-4 VP7, BoHV-4 VP2, and BoHV-4 VP2 + VP7 vaccinated mice. Representative dot-plots of unstimulated cells from BoHV-4 VP7 responding mice (i.e., background production) are shown as control. [file 13567_2026_1771_MOESM1_ESM.tif]

pINT2-CMV-VP7-HA

Phase contrast

DAPI

Anti-HA

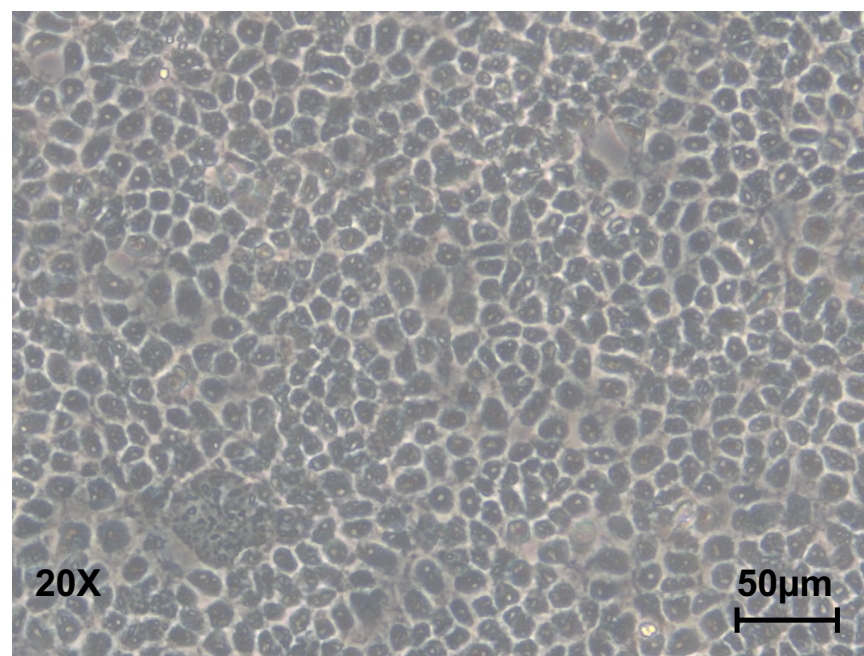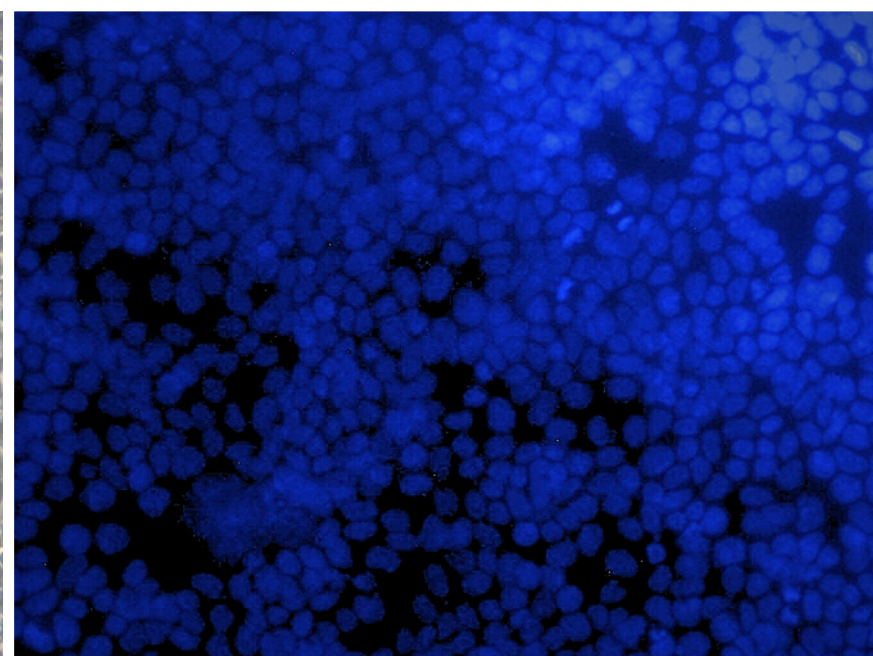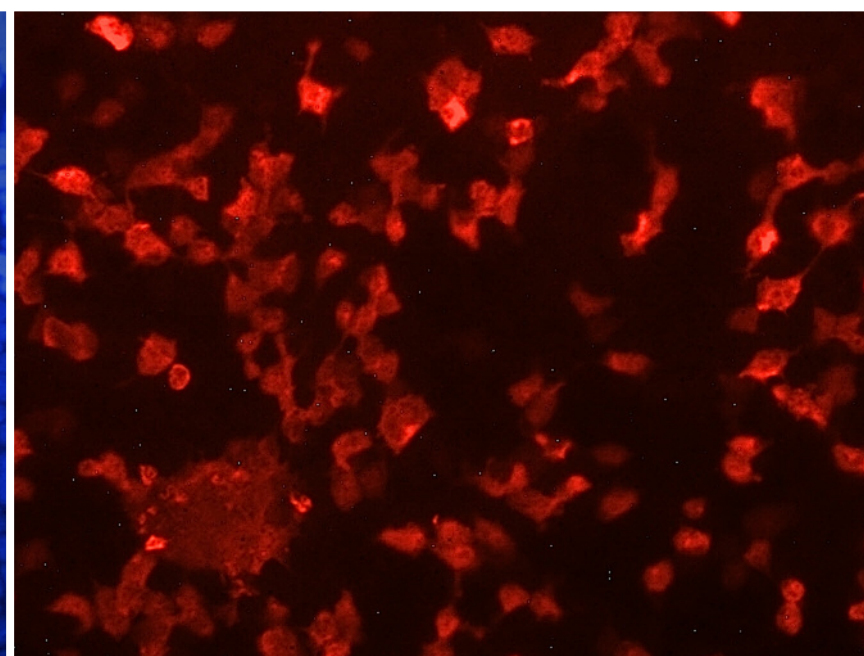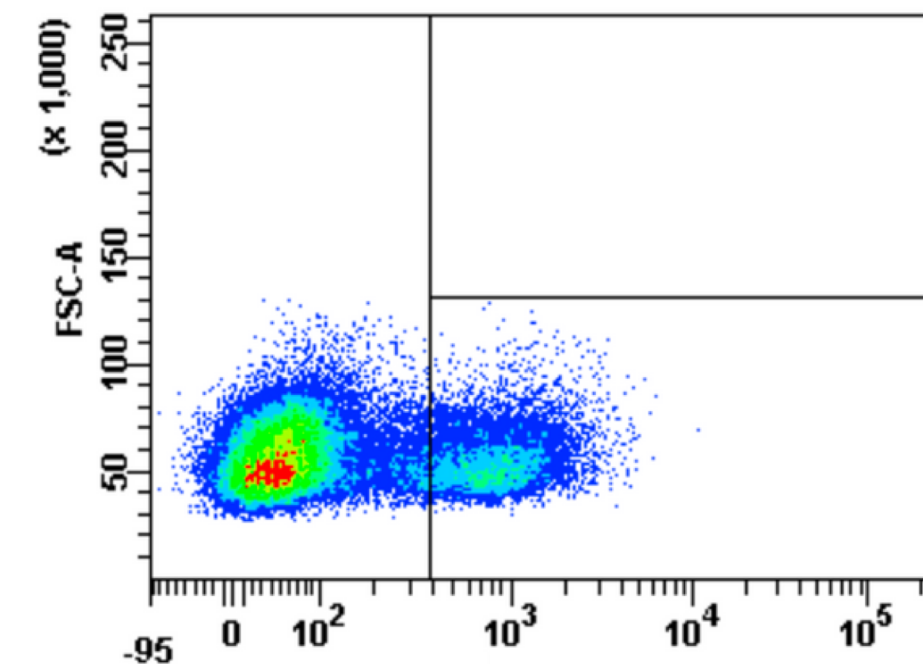

Control

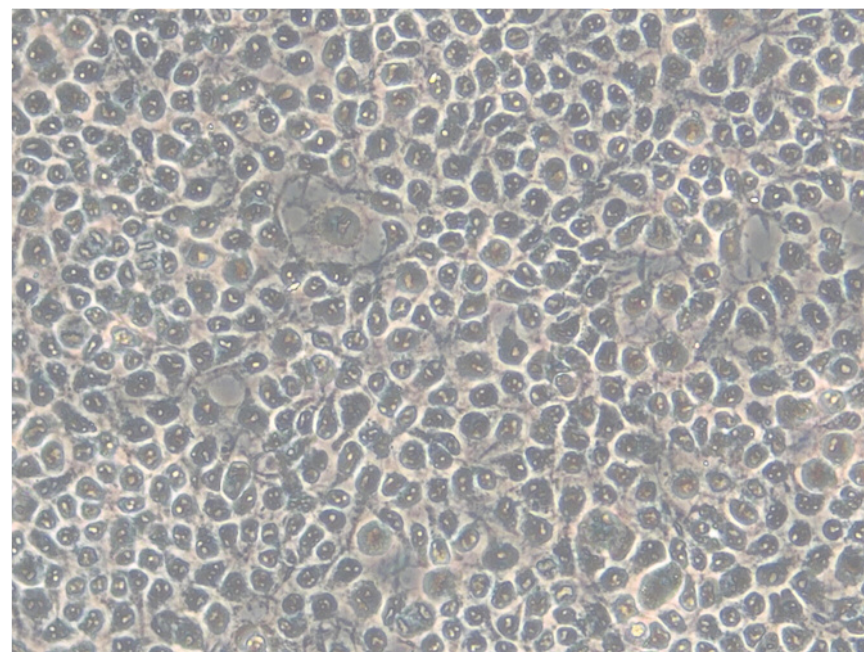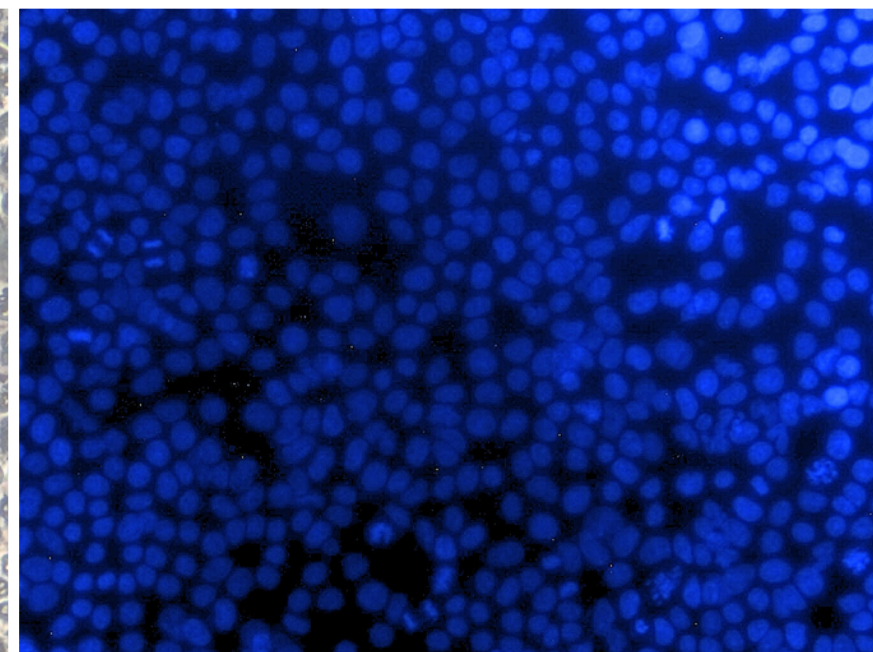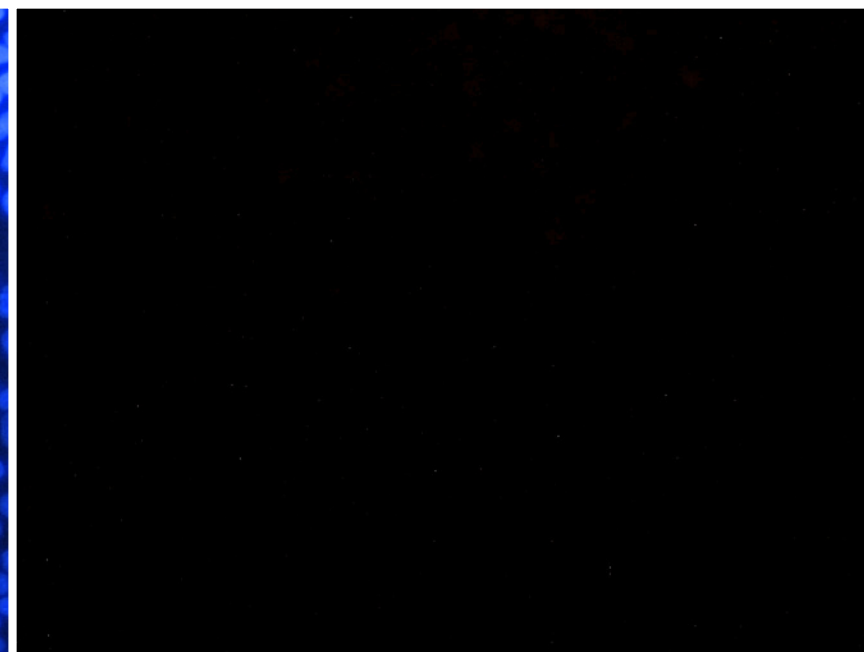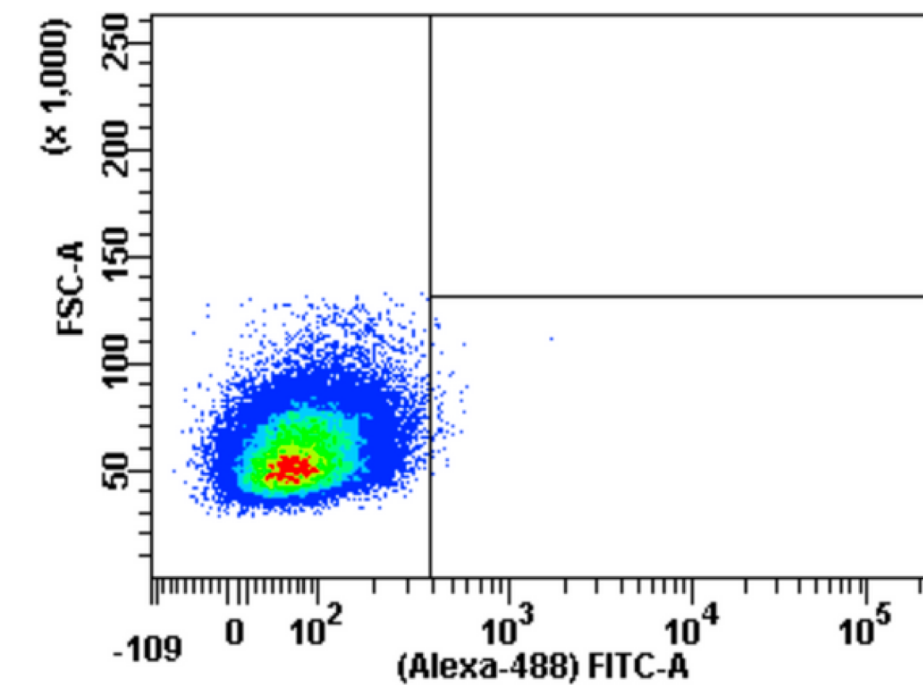

Supplement: Supplementary file 2 — Additional file 2. Staining of pINT2-(TK-CMV-VP7-HA-TK)-transfected HEK293 cells and stained with an anti-HA mAb antibody and analyzed by immunofluorescence and flow cytometry. [file 13567_2026_1771_MOESM2_ESM.pdf]
